# Effectiveness and safety of SARS-CoV-2 vaccine in real-world studies: a systematic review and meta-analysis

**DOI:** 10.1186/s40249-021-00915-3

**Published:** 2021-11-14

**Authors:** Qiao Liu, Chenyuan Qin, Min Liu, Jue Liu

**Affiliations:** 1 Department of Epidemiology and Biostatistics, School of Public Health, Peking University, Beijing, 100191 China; 2 Institute for Global Health and Development, Peking University, Beijing, 100871 China

**Keywords:** SARS-CoV-2, Vaccine, Effectiveness, Safety, Meta-analysis

## Abstract

**Background:**

To date, coronavirus disease 2019 (COVID-19) becomes increasingly fierce due to the emergence of variants. Rapid herd immunity through vaccination is needed to block the mutation and prevent the emergence of variants that can completely escape the immune surveillance. We aimed to systematically evaluate the effectiveness and safety of COVID-19 vaccines in the real world and to establish a reliable evidence-based basis for the actual protective effect of the COVID-19 vaccines, especially in the ensuing waves of infections dominated by variants.

**Methods:**

We searched PubMed, Embase and Web of Science from inception to July 22, 2021. Observational studies that examined the effectiveness and safety of SARS-CoV-2 vaccines among people vaccinated were included. Random-effects or fixed-effects models were used to estimate the pooled vaccine effectiveness (VE) and incidence rate of adverse events after vaccination, and their 95% confidence intervals (*CI*).

**Results:**

A total of 58 studies (32 studies for vaccine effectiveness and 26 studies for vaccine safety) were included. A single dose of vaccines was 41% (95% *CI*: 28–54%) effective at preventing SARS-CoV-2 infections, 52% (31–73%) for symptomatic COVID-19, 66% (50–81%) for hospitalization, 45% (42–49%) for Intensive Care Unit (ICU) admissions, and 53% (15–91%) for COVID-19-related death; and two doses were 85% (81–89%) effective at preventing SARS-CoV-2 infections, 97% (97–98%) for symptomatic COVID-19, 93% (89–96%) for hospitalization, 96% (93–98%) for ICU admissions, and 95% (92–98%) effective for COVID-19-related death, respectively. The pooled VE was 85% (80–91%) for the prevention of Alpha variant of SARS-CoV-2 infections, 75% (71–79%) for the Beta variant, 54% (35–74%) for the Gamma variant, and 74% (62–85%) for the Delta variant. The overall pooled incidence rate was 1.5% (1.4–1.6%) for adverse events, 0.4 (0.2–0.5) per 10 000 for severe adverse events, and 0.1 (0.1–0.2) per 10 000 for death after vaccination.

**Conclusions:**

SARS-CoV-2 vaccines have reassuring safety and could effectively reduce the death, severe cases, symptomatic cases, and infections resulting from SARS-CoV-2 across the world. In the context of global pandemic and the continuous emergence of SARS-CoV-2 variants, accelerating vaccination and improving vaccination coverage is still the most important and urgent matter, and it is also the final means to end the pandemic.

**Graphical Abstract:**

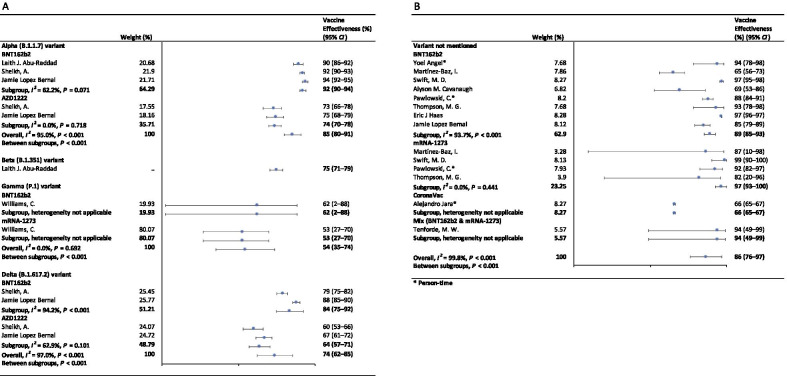

**Supplementary Information:**

The online version contains supplementary material available at 10.1186/s40249-021-00915-3.

## Background

Since its outbreak, coronavirus disease 2019 (COVID-19) has spread rapidly, with a sharp rise in the accumulative number of infections worldwide. As of August 8, 2021, COVID-19 has already killed more than 4.2 million people and more than 203 million people were infected [1]. Given its alarming-spreading speed and the high cost of completely relying on non-pharmaceutical measures, we urgently need safe and effective vaccines to cover susceptible populations and restore people’s lives into the original [2].

According to global statistics, as of August 2, 2021, there are 326 candidate vaccines, 103 of which are in clinical trials, and 19 vaccines have been put into normal use, including 8 inactivated vaccines and 5 protein subunit vaccines, 2 RNA vaccines, as well as 4 non-replicating viral vector vaccines [3]. Our World in Data simultaneously reported that 27.3% of the world population has received at least one dose of a COVID-19 vaccine, and 13.8% is fully vaccinated [4].

To date, COVID-19 become increasingly fierce due to the emergence of variants [5–7]. Rapid herd immunity through vaccination is needed to block the mutation and prevent the emergence of variants that can completely escape the immune surveillance [6, 8]. Several reviews systematically evaluated the effectiveness and/or safety of the three mainstream vaccines on the market (inactivated virus vaccines, RNA vaccines and viral vector vaccines) based on random clinical trials (RCT) yet [9–13].

In general, RNA vaccines are the most effective, followed by viral vector vaccines and inactivated virus vaccines [10–13]. The current safety of COVID-19 vaccines is acceptable for mass vaccination, but long-term monitoring of vaccine safety is needed, especially in older people with underlying conditions [9–13]. Inactivated vaccines had the lowest incidence of adverse events and the safety comparisons between mRNA vaccines and viral vectors were controversial [9, 10].

RCTs usually conduct under a very demanding research circumstance, and tend to be highly consistent and limited in terms of population characteristics and experimental conditions. Actually, real-world studies differ significantly from RCTs in terms of study conditions and mass vaccination in real world requires taking into account factors, which are far more complex, such as widely heterogeneous populations, vaccine supply, willingness, medical accessibility, etc. Therefore, the real safety and effectiveness of vaccines turn out to be a major concern of international community. The results of a mass vaccination of CoronaVac in Chile demonstrated a protective effectiveness of 65.9% against the onset of COVID-19 after complete vaccination procedures [14], while the outcomes of phase 3 trials in Brazil and Turkey were 50.7% and 91.3%, reported on Sinovac’s website [14]. As for the Delta variant, the British claimed 88% protection after two doses of BNT162b2, compared with 67% for AZD1222 [15]. What is surprising is that the protection of BNT162b2 against infection in Israel is only 39% [16]. Several studies reported the effectiveness and safety of the COVID-19 vaccine in the real world recently, but the results remain controversial [17–20]. A comprehensive meta-analysis based upon the real-world studies is still in an urgent demand, especially for evaluating the effect of vaccines on variation strains. In the present study, we aimed to systematically evaluate the effectiveness and safety of the COVID-19 vaccine in the real world and to establish a reliable evidence-based basis for the actual protective effect of the COVID-19 vaccines, especially in the ensuing waves of infections dominated by variants.

## Methods

### Search strategy and selection criteria

Our methods were described in detail in our published protocol [PROSPERO (Prospective register of systematic reviews) registration, CRD42021267110]. We searched eligible studies published by 22 July 2021, from three databases including PubMed, Embase and Web of Science by the following search terms: (effectiveness OR safety) AND (COVID-19 OR coronavirus OR SARS-CoV-2) AND (vaccine OR vaccination). We used EndNoteX9.0 (Thomson ResearchSoft, Stanford, USA) to manage records, screen and exclude duplicates. This study was strictly performed according to the Preferred Reporting Items for Systematic Reviews and Meta-Analyses (PRISMA).

We included observational studies that examined the effectiveness and safety of severe acute respiratory syndrome coronavirus 2 (SARS-CoV-2) vaccines among people vaccinated with SARS-CoV-2 vaccines. The following studies were excluded: (1) irrelevant to the subject of the meta-analysis, such as studies that did not use SARS-CoV-2 vaccination as the exposure; (2) insufficient data to calculate the rate for the prevention of COVID-19, the prevention of hospitalization, the prevention of admission to the ICU, the prevention of COVID-19-related death, or adverse events after vaccination; (3) duplicate studies or overlapping participants; (4) RCT studies, reviews, editorials, conference papers, case reports or animal experiments; and (5) studies that did not clarify the identification of COVID-19.

Studies were identified by two investigators (LQ and QCY) independently following the criteria above, while discrepancies reconciled by a third investigator (LJ).

### Data extraction and quality assessment

The primary outcome was the effectiveness of SARS-CoV-2 vaccines. The following data were extracted independently by two investigators (LQ and QCY) from the selected studies: (1) basic information of the studies, including first author, publication year and study design; (2) characteristics of the study population, including sample sizes, age groups, setting or locations; (3) kinds of the SARS-CoV-2 vaccines; (4) outcomes for the effectiveness of SARS-CoV-2 vaccines: the number of laboratory-confirmed COVID-19, hospitalization for COVID-19, admission to the ICU for COVID-19, and COVID-19-related death; and (5) outcomes for the safety of SARS-CoV-2 vaccines: the number of adverse events after vaccination.

We evaluated the risk of bias using the Newcastle–Ottawa quality assessment scale for cohort studies and case–control studies [21]. and assess the methodological quality using the checklist recommended by Agency for Healthcare Research and Quality (AHRQ) [22]. Cohort studies and case–control studies were classified as having low (≥ 7 stars), moderate (5–6 stars), and high risk of bias (≤ 4 stars) with an overall quality score of 9 stars. For cross-sectional studies, we assigned each item of the AHRQ checklist a score of 1 (answered “yes”) or 0 (answered “no” or “unclear”), and summarized scores across items to generate an overall quality score that ranged from 0 to 11. Low, moderate, and high risk of bias were identified as having a score of 8–11, 4–7 and 0–3, respectively.

Two investigators (LQ and QCY) independently assessed study quality, with disagreements resolved by a third investigator (LJ).

### Data synthesis and statistical analysis

We performed a meta-analysis to pool data from included studies and assess the effectiveness and safety of SARS-CoV-2 vaccines by clinical outcomes (rates of the prevention of COVID-19, the prevention of hospitalization, the prevention of admission to the ICU, the prevention of COVID-19-related death, and adverse events after vaccination). Random-effects or fixed-effects models were used to pool the rates and adjusted estimates across studies separately, based on the heterogeneity between estimates (*I*^2^). Fixed-effects models were used if *I*^2^ ≤ 50%, which represented low to moderate heterogeneity and random-effects models were used if *I*^2^ > 50%, representing substantial heterogeneity.

We conducted subgroup analyses to investigate the possible sources of heterogeneity by using vaccine kinds, vaccination status, sample size, and study population as grouping variables. We used the *Q* test to conduct subgroup comparisons and variables were considered significant between subgroups if the subgroup difference *P* value was less than 0.05. Publication bias was assessed by funnel plot and Egger’s regression test. We analyzed data using Stata version 16.0 (StataCorp, Texas, USA).

## Results

**Fig. 1:**
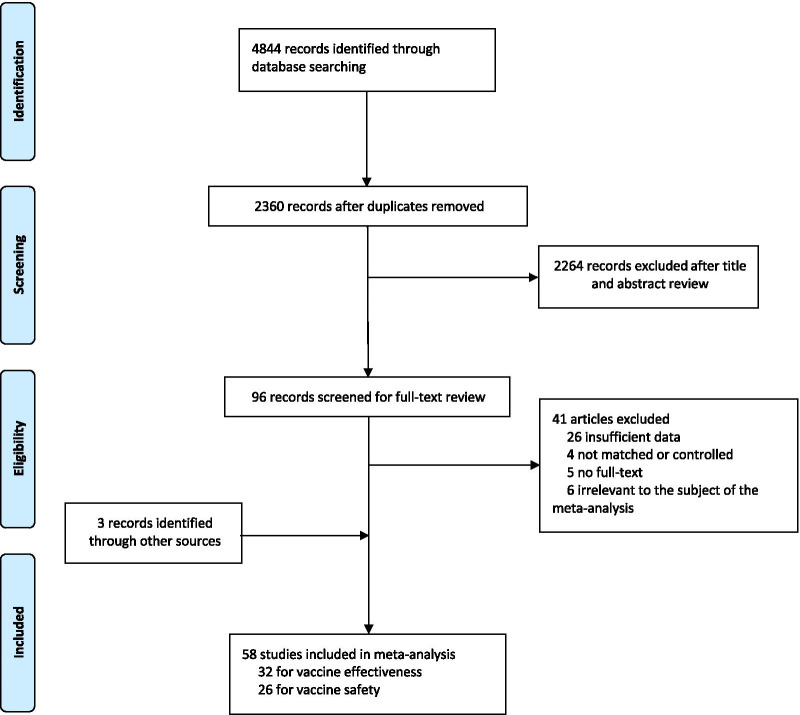
Flowchart of the study selection

A total of 4844 records were searched from the three databases. 2484 duplicates were excluded. After reading titles and abstracts, we excluded 2264 reviews, RCT studies, duplicates and other studies meeting our exclude criteria. Among the 96 studies under full-text review, 41 studies were excluded (Fig. 1). Ultimately, with three grey literatures included, this final meta-analysis comprised 58 eligible studies, including 32 studies [14, 15, 17–20, 23–48] for vaccine effectiveness and 26 studies [49–74] for vaccine safety. Characteristics of included studies are showed in Additional file 1: Table S1, Additional file 2: Table S2. The risk of bias of all studies we included was moderate or low.

### Vaccine effectiveness for different clinical outcomes of COVID-19

We separately reported the vaccine effectiveness (VE) by the first and second dose of vaccines, and conducted subgroup analysis by the days after the first or second dose (< 7 days, ≥ 7 days, ≥ 14 days, and ≥ 21 days; studies with no specific days were classified as 1 dose, 2 dose or ≥ 1 dose).

For the first dose of SARS-CoV-2 vaccines, the pooled VE was 41% (95% *CI*: 28–54%) for the prevention of SARS-CoV-2 infection, 52% (95% *CI*: 31–73%) for the prevention of symptomatic COVID-19, 66% (95% *CI*: 50–81%) for the prevention of hospital admissions, 45% (95% *CI*: 42–49%) for the prevention of ICU admissions, and 53% (95% *CI*: 15–91%) for the prevention of COVID-19-related death (Table 1). The subgroup, ≥ 21 days after the first dose, was found to have the highest VE in each clinical outcome of COVID-19, regardless of ≥ 1 dose group (Table 1).

**Table 1:** Effectiveness of a single dose and two doses of SARS-CoV-2 vaccines

	No. cohorts	Vaccine effectiveness (%) (95% CI)	*I*^2^ (%)	*P* value for heterogeneity	*P* value for subgroup differences	Weight (%)
The first dose						
*Prevention of SARS-CoV-2 infection*						
Overall	44	41 (28–54)	99.6	< 0.001	< 0.001	100
< 7 days after the first dose	3	1 (− 25–27)	84.0	0.002		6.92
< 14 days after the first dose	4	12 (0–24)	0.0	0.759		6.78
≥ 7 days after the first dose	5	17 (− 20–55)	95.3	< 0.001		10.61
≥ 14 days after the first dose	20	48 (31–66)	99.4	< 0.001		46.63
≥ 21 days after the first dose	8	56 (42–70)	92.6	< 0.001		19.45
1 dose	3	32 (9–54)	83.4	0.002		7.11
≥ 1 dose	1	95 (94–96)	..	..		2.50
*Prevention of symptomatic COVID-19*						
Overall	14	52 (31–73)	99.2	< 0.001	< 0.001	100
< 7 days after the first dose	1	19 (0–45)	..	..		6.79
≥ 7 days after the first dose	2	45 (− 43–100)	99.8	< 0.001		14.63
≥ 14 days after the first dose	5	53 (43–62)	64.2	0.025		35.24
≥ 21 days after the first dose	2	63 (7–100)	99.1	< 0.001		14.56
1 dose	3	44 (14–74)	94.4	< 0.001		21.43
≥ 1 dose	1	95 (92–97)	..	..		7.34
*Prevention of COVID-19 hospital admissions*						
Overall	8	66 (50–81)	98.5	< 0.001	0.005	100
< 7 days after the first dose	1	74 (71–77)	..	..		13.47
≥ 7 days after the first dose	1	61 (47–75)	..	..		12.27
≥ 14 days after the first dose	5	61 (41–81)	97.9	< 0.001		60.93
≥ 21 days after the first dose	1	82 (77–88)	..	..		13.33
*Prevention of COVID-19 ICU admissions*						
Overall	3	45 (42–49)	39.5	0.192	..	100
≥ 14 days after the first dose	3	45 (42–49)	39.5	0.192		100
*Prevention of COVID-19-related death*						
Overall	4	53 (15–91)	98.9	< 0.001	< 0.001	100
≥ 14 days after the first dose	3	44 (23–64)	58.1	0.092		70.60
≥ 1 dose	1	97 (91–99)	..	..		29.40
The second dose						
*Prevention of SARS-CoV-2 infection*						
Overall	34	85 (81–89)	99.5	< 0.001	< 0.001	100
< 7 days after the second dose	3	75 (51–99)	95.7	< 0.001		8.72
< 14 days after the second dose	2	70 (49–91)	70.8	0.064		4.77
≥ 7 days after the second dose	14	91 (88–93)	76.5	< 0.001		42.72
≥ 14 days after the second dose	13	81 (71–92)	99.8	< 0.001		37.35
≥ 21 days after the second dose	1	94 (78–98)	..	..		2.91
2 doses	1	98 (96–99)	..	..		3.52
*Prevention of symptomatic COVID-19*						
Overall	17	97 (97–98)	83.6	< 0.001	0.008	100
< 7 days after the second dose	1	100 (94–100)	..	..		5.53
< 14 days after the second dose	2	75 (59–92)	46.5	0.171		0.47
≥ 7 days after the second dose	5	97 (96–98)	17.4	0.304		32.25
≥ 14 days after the second dose	7	96 (94–98)	87.9	< 0.001		46.53
≥ 21 days after the second dose	1	99 (94–100)	..	..		5.67
2 doses	1	99 (96–100)	..	..		9.54
*Prevention of COVID-19 hospital admissions*						
Overall	8	93 (89–96)	99.0	< 0.001	0.991	100
≥ 7 days after the second dose	3	93 (84–100)	72.5	0.026		35.70
≥ 14 days after the second dose	5	93 (85–100)	99.4	< 0.001		64.3
*Prevention of COVID-19 ICU admissions*						
Overall	8	96 (93–98)	96.4	< 0.001	0.414	100
≥ 7 days after the second dose	4	97 (93–100)	18.2	0.300		38.11
≥ 14 days after the second dose	4	94 (87–100)	98.4	< 0.001		61.89
*Prevention of COVID-19-related death*						
Overall	8	95 (92–98)	96.3	< 0.001	0.851	100
< 14 days after the second dose	1	96 (83–99)	..	..		8.68
≥ 7 days after the second dose	3	97 (95–98)	11.7	0.322		37.91
≥ 14 days after the second dose	3	93 (82–100)	98.9	< 0.001		42.36
2 doses	1	98 (87–100)	..	..		11.06

*CI* confidence interval, *SARS-CoV-2* severe acute respiratory syndrome coronavirus 2, *COVID-19* coronavirus disease 2019

For the second dose of SARS-CoV-2 vaccines, the pooled VE was 85% (95% *CI*: 81–89%) for the prevention of SARS-CoV-2 infection, 97% (95% *CI*: 97–98%) for the prevention of symptomatic COVID-19, 93% (95% CI: 89–96%) for the prevention of hospital admissions, 96% (95%* CI*: 93–98%) for the prevention of ICU admissions, and 95% (95% *CI*: 92–98%) for the prevention of COVID-19-related death (Table 1). VE was 94% (95% *CI*: 78–98%) in ≥ 21 days after the second dose for the prevention of SARS-CoV-2 infection, higher than other subgroups, regardless of 2 dose group (Table 1). For the prevention of symptomatic COVID-19, VE was also relatively higher in 21 days after the second dose (99%, 95% *CI*: 94–100%). Subgroups showed no statistically significant differences in the prevention of hospital admissions, ICU admissions and COVID-19-related death (subgroup difference *P* values were 0.991, 0.414, and 0.851, respectively).

### Vaccine effectiveness for different variants of SARS-CoV-2 in fully vaccinated people

**Fig. 2:**
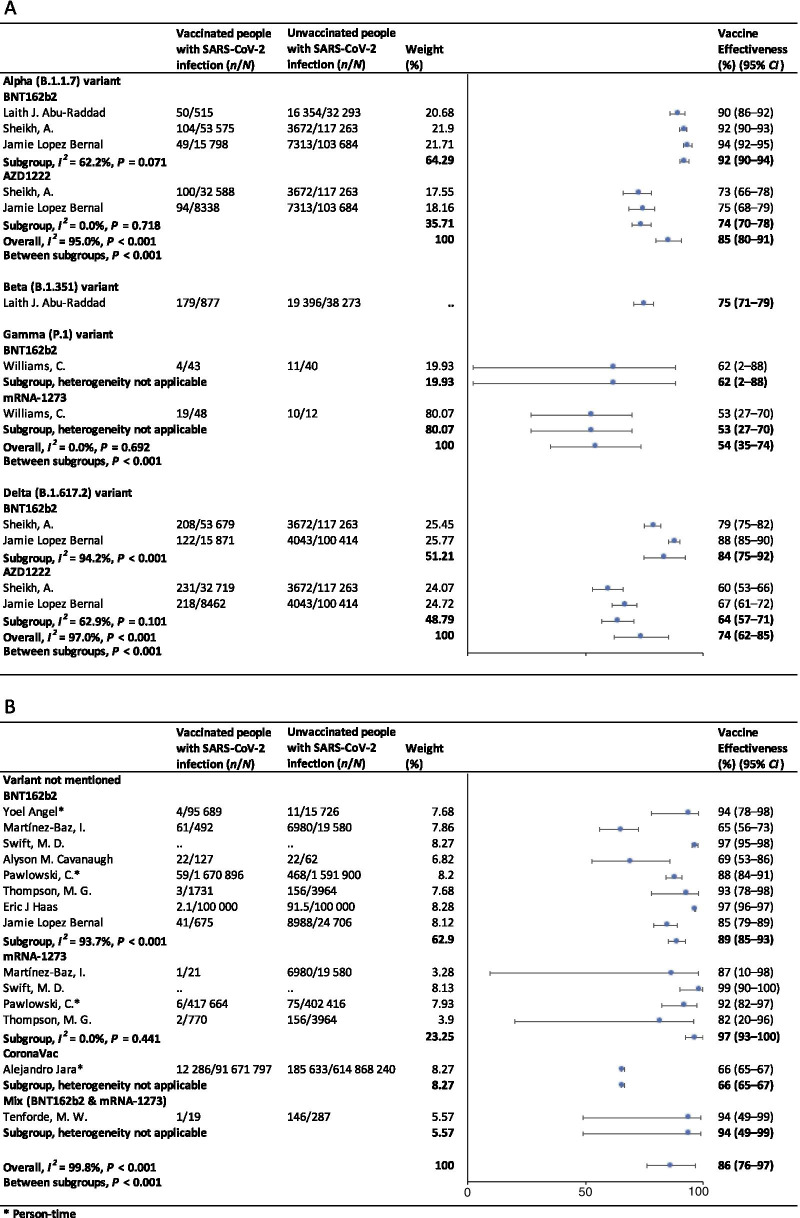
Forest plots for the vaccine effectiveness of SARS-CoV-2 vaccines in fully vaccinated populations. **A** Vaccine effectiveness against SARS-CoV-2 variants; **B** Vaccine effectiveness against SARS-CoV-2 with variants not mentioned. *SARS-CoV-2* severe acute respiratory syndrome coronavirus 2, *COVID-19* coronavirus disease 2019, *CI* confidence interval

In the fully vaccinated groups (over 14 days after the second dose), the pooled VE was 85% (95% CI: 80–91%) for the prevention of Alpha variant of SARS-CoV-2 infection, 54% (95% *CI*: 35–74%) for the Gamma variant, and 74% (95% *CI*: 62–85%) for the Delta variant. There was only one study [23] focused on the Beta variant, which showed the VE was 75% (95% *CI*: 71–79%) for the prevention of the Beta variant of SARS-CoV-2 infection. BNT162b2 vaccine had the highest VE in each variant group; 92% (95% *CI*: 90–94%) for the Alpha variant, 62% (95% *CI*: 2–88%) for the Gamma variant, and 84% (95% *CI*: 75–92%) for the Delta variant (Fig. 2).

For studies which had not mentioned the variant of SARS-CoV-2, the pooled VE was 86% (95% CI: 76–97%) for the prevention of SARS-CoV-2 infection in fully vaccinated people. mRNA-1273 vaccine had the highest pooled VE (97%, 95% CI: 93–100%, Fig. 2).

### Safety of SARS-CoV-2 vaccines

As Table 2 showed, the incidence rate of adverse events varied widely among different studies. We conducted subgroup analysis by study population (general population, patients and healthcare workers), vaccine type (BNT162b2, mRNA-1273, CoronaVac, and et al.), and population size (< 1000, 1000–10 000, 10 000–100 000, and > 100 000). The overall pooled incidence rate was 1.5% (95% *CI*: 1.4–1.6%) for adverse events, 0.4 (95% *CI*: 0.2–0.5) per 10 000 for severe adverse events, and 0.1 (95% *CI*: 0.1–0.2) per 10 000 for death after vaccination. Incidence rate of adverse events was higher in healthcare workers (53.2%, 95% *CI*: 28.4–77.9%), AZD1222 vaccine group (79.6%, 95% *CI*: 60.8–98.3%), and < 1000 population size group (57.6%, 95% *CI*: 47.9–67.4%). Incidence rate of sever adverse events was higher in healthcare workers (127.2, 95% *CI*: 62.7–191.8, per 10 000), Gam-COVID-Vac vaccine group (175.7, 95% *CI*: 77.2–274.2, per 10 000), and 1000–10 000 population size group (336.6, 95% *CI*: 41.4–631.8, per 10 000). Incidence rate of death after vaccination was higher in patients (7.6, 95% *CI*: 0.0–32.2, per 10 000), BNT162b2 vaccine group (29.8, 95% *CI*: 0.0–71.2, per 10 000), and < 1000 population size group (29.8, 95%* CI*: 0.0–71.2, per 10 000). Subgroups of general population, vaccine type not mentioned, and > 100 000 population size had the lowest incidence rate of adverse events, severe adverse events, and death after vaccination.

**Table 2:** Subgroup analysis of incidence rate of adverse events, sever adverse events and death after SARS-CoV-2 vaccination, by study population, vaccine type, and population size

	No. cohorts	Incidence rate of adverse events (%) (95% CI)	*I*^2^ (%)	*P* value for heterogeneity	*P* value for subgroup differences	Weight (%)
Overall	31	1.5 (1.4–1.6)	100.0	< 0.001		100
*Study population*					< 0.001	
General population	10	1.0 (0.9–1.0)	100.0	< 0.001		98.92
Healthcare workers	6	53.2 (28.4–77.9)	99.8	< 0.001		0.80
Patients	15	45.7 (33.8–57.6)	97.2	< 0.001		0.28
*Vaccine type*					< 0.001	
BNT162b2	17	49.6 (21.8–77.4)	99.9	< 0.001		23.57
AZD1222	4	79.6 (60.8–98.3)	99.1	< 0.001		0.49
mRNA-1273	1	50.0 (34.9–65.1)	..	..		0.01
CoronaVac	3	30.8 (7.8–53.8)	99.7	< 0.001		0.63
Ad26.COV2.S	1	0.2 (0.2–0.2)	..	..		25.03
Gam-COVID-Vac	1	71.3 (67.9–74.7)	..	..		0.08
BBIBP-CorV	1	29.4 (24.8–34.0)	..	..		0.04
Any	3	0.0 (0.0–0.1)	100.0	< 0.001		50.15
*Population size*					< 0.001	
< 1000	24	57.6 (47.9–67.4)	99.2	< 0.001		1.41
1000–10 000	3	29.1 (3.3–54.9)	99.8	< 0.001		0.75
10 000–100 000	1	0.4 (0.3–0.4)	..	..		22.74
> 100 000	3	0.1 (0.0–0.1)	100.0	< 0.001		75.11

	No. cohorts	Incidence rate of severe adverse events (per 10 000) (95% CI)	*I*^2^ (%)	*P* value for heterogeneity	*P* value for subgroup differences	Weight (%)
Overall	15	0.4 (0.2–0.5)	98.2	< 0.001		100
*Study population*					< 0.001	
General population	9	0.4 (0.2–0.5)	98.9	< 0.001		84.53
Healthcare workers	2	127.2 (62.7–191.8)	36.1	0.211		0.01
Patients	4	22.5 (0.0–72.6)	77.7	0.004		15.46
*Vaccine type*					< 0.001	
BNT162b2	5	8.1 (0.0–17.8)	98.2	< 0.001		7.71
mRNA-1273	2	4.2 (0.0–16.3)	43.9	0.182		0.83
CoronaVac	1	104.8 (53.7–156.0)	..	..		0.01
Ad26.COV2.S	1	0.4 (0.4–0.5)	..	..		18.84
Gam-COVID-Vac	1	175.7 (77.2–274.2)	..	..		0.01
Any	5	0.3 (0.2–0.5)	99.1	< 0.001		72.60
*Population size*					0.030	
< 1000	4	90.4 (0.0–193.5)	82.7	0.001		0.01
1000–10 000	3	336.6 (41.4–631.8)	99.0	< 0.001		0.01
10 000–100 000	3	1.0 (0.0–2.2)	73.3	0.024		8.54
> 100 000	5	0.4 (0.2–0.5)	99.2	< 0.001		91.45

	No. cohorts	Incidence rate of death after vaccination (per 10 000) (95% CI)	*I*^2^ (%)	*P* value for heterogeneity	*P* value for subgroup differences	Weight (%)
Overall	5	0.1 (0.1–0.2)	87.6	< 0.001		100
*Study population*					0.549	
General population	3	0.1 (0.1–0.1)	55.4	0.106		94.73
Patients	2	7.6 (0.0–32.2)	48.3	0.164		5.27
*Vaccine type*					0.319	
BNT162b2	1	29.8 (0.0–71.2)	..	..		0.01
Ad26.COV2.S	1	0.1 (0.1–0.1)	..	..		30.92
Any	3	0.1 (0.1–0.2)	92.4	< 0.001		69.07
*Population size*					0.158	
< 1000	1	29.8 (0.0–71.2)	..	..		0.01
> 100 000	4	0.1 (0.1–0.2)	90.0	< 0.001		99.9

*CI* confidence interval

### Sensitivity analysis and publication bias

In the sensitivity analyses, VE for SARS-CoV-2 infections, symptomatic COVID-19 and COVID-19-related death got relatively lower when omitting over a single dose group of Maria et al.’s work [33]; when omitting ≥ 14 days after the first dose group and ≥ 14 days after the second dose group of Alejandro et al.’s work [14], VE for SARS-CoV-2 infections, hospitalization, ICU admission and COVID-19-related death got relatively higher; and VE for all clinical status of COVID-19 became lower when omitting ≥ 14 days after the second dose group of Eric et al.’s work [34]. Incidence rate of adverse events and severe adverse events got relatively higher when omitting China CDC’s data [74]. *P* values of Egger’s regression test for all the meta-analysis were more than 0.05, indicating that there might not be publication bias.

## Discussion

To our knowledge, this is a comprehensive systematic review and meta-analysis assessing the effectiveness and safety of SARS-CoV-2 vaccines based on real-world studies, reporting pooled VE for different variants of SARS-CoV-2 and incidence rate of adverse events. This meta-analysis comprised a total of 58 studies, including 32 studies for vaccine effectiveness and 26 studies for vaccine safety. We found that a single dose of SARS-CoV-2 vaccines was about 40–60% effective at preventing any clinical status of COVID-19 and that two doses were 85% or more effective. Although vaccines were not as effective against variants of SARS-CoV-2 as original virus, the vaccine effectiveness was still over 50% for fully vaccinated people. Normal adverse events were common, while the incidence of severe adverse events or even death was very low, providing reassurance to health care providers and to vaccine recipients and promote confidence in the safety of COVID-19 vaccines. Our findings strengthen and augment evidence from previous review [75], which confirmed the effectiveness of the BNT162b2 mRNA vaccine, and additionally reported the safety of SARS-CoV-2 vaccines, giving insight on the future of SARS-CoV-2 vaccine schedules.

Although most vaccines for the prevention of COVID-19 are two-dose vaccines, we found that the pooled VE of a single dose of SARS-CoV-2 vaccines was about 50%. Recent study showed that the T cell and antibody responses induced by a single dose of the BNT162b2 vaccine were comparable to those naturally infected with SARE-CoV-2 within weeks or months after infection [76]. Our findings could help to develop vaccination strategies under certain circumstances such as countries having a shortage of vaccines. In some countries, in order to administer the first dose to a larger population, the second dose was delayed for up to 12 weeks [77]. Some countries such as Canada had even decided to delay the second dose for 16 weeks [78]. However, due to a suboptimum immune response in those receiving only a single dose of a vaccine, such an approach had a chance to give rise to the emergence of variants of SARS-CoV-2 [79]. There remains a need for large clinical trials to assess the efficacy of a single-dose administration of two-dose vaccines and the risk of increasing the emergence of variants.

Two doses of SARS-CoV-2 vaccines were highly effective at preventing hospitalization, severe cases and deaths resulting from COVID-19, while the VE of different groups of days from the second vaccine dose showed no statistically significant differences. Our findings emphasized the importance of getting fully vaccinated, for the fact that most breakthrough infections were mild or asymptomatic. A recent study showed that the occurrence of breakthrough infections with SARS-CoV-2 in fully vaccinated populations was predictable with neutralizing antibody titers during the peri-infection period [80]. We also found getting fully vaccinated was at least 50% effective at preventing SARS-CoV-2 variants infections, despite reduced effectiveness compared with original virus; and BNT162b2 vaccine was found to have the highest VE in each variant group. Studies showed that the highly mutated variants were indicative of a form of rapid, multistage evolutionary jumps, which could preferentially occur in the milieu of partial immune control [81, 82]. Therefore, immunocompromised patients should be prioritized for anti-COVID-19 immunization to mitigate persistent SARS-CoV-2 infections, during which multimutational SARS-CoV-2 variants could arise [83].

Recently, many countries, including Israel, the United States, China and the United Kingdom, have introduced a booster of COVID-19 vaccine, namely the third dose [84–87]. A study of Israel showed that among people vaccinated with BNT162b2 vaccine over 60 years, the risk of COVID-19 infection and severe illness in the non-booster group was 11.3 times (95% CI: 10.4–12.3) and 19.5 times (95% CI: 12.9–29.5) than the booster group, respectively [84]. Some studies have found that the third dose of Moderna, Pfizer-BioNTech, Oxford-AstraZeneca and Sinovac produced a spike in infection-blocking neutralizing antibodies when given a few months after the second dose [85, 87, 88]. In addition, the common adverse events associated with the third dose did not differ significantly from the symptoms of the first two doses, ranging from mild to moderate [85]. The overall incidence rate of local and systemic adverse events was 69% (57/97) and 20% (19/97) after receiving the third dose of BNT162b2 vaccine, respectively [88]. Results of a phase 3 clinical trial involving 306 people aged 18–55 years showed that adverse events after receiving a third dose of BNT162b2 vaccine (5–8 months after completion of two doses) were similar to those reported after receiving a second dose [85]. Based on V-safe, local reactions were more frequently after dose 3 (5323/6283; 84.7%) than dose 2 (5249/6283; 83.5%) among people who received 3 doses of Moderna. Systemic reactions were reported less frequently after dose 3 (4963/6283; 79.0%) than dose 2 (5105/6283; 81.3%) [86]. On August 4, WHO called for a halt to booster shots until at least the end of September to achieve an even distribution of the vaccine [89]. At this stage, the most important thing we should be thinking about is how to reach a global cover of people at risk with the first or second dose, rather than focusing on the third dose.

Based on real world studies, our results preliminarily showed that complete inoculation of COVID-19 vaccines was still effective against infection of variants, although the VE was generally diminished compared with the original virus. Particularly, the pooled VE was 54% (95%* CI*: 35–74%) for the Gamma variant, and 74% (95% *CI*: 62–85%) for the Delta variant. Since the wide spread of COVID-19, a number of variants have drawn extensive attention of international community, including Alpha variant (B.1.1.7), first identified in the United Kingdom; Beta variant (B.1.351) in South Africa; Gamma variant (P.1), initially appeared in Brazil; and the most infectious one to date, Delta variant (B.1.617.2) [90]. Israel recently reported a breakthrough infection of SARS-CoV-2, dominated by variant B.1.1.7 in a small number of fully vaccinated health care workers, raising concerns about the effectiveness of the original vaccine against those variants [80]. According to an observational cohort study in Qatar, VE of the BNT162b2 vaccine against the Alpha (B.1.1.7) and Beta (B.1.351) variants was 87% (95% *CI*: 81.8–90.7%) and 75.0% (95% *CI*: 70.5–7.9%), respectively [23]. Based on the National Immunization Management System of England, results from a recent real-world study of all the general population showed that the AZD1222 and BNT162b2 vaccines protected against symptomatic SARS-CoV-2 infection of Alpha variant with 74.5% (95% *CI*: 68.4–79.4%) and 93.7% (95% *CI*: 91.6–95.3%) [15]. In contrast, the VE against the Delta variant was 67.0% (95% *CI*: 61.3–71.8%) for two doses of AZD1222 vaccine and 88% (95% *CI*: 85.3–90.1%) for BNT162b2 vaccine [15].

In terms of adverse events after vaccination, the pooled incidence rate was very low, only 1.5% (95% *CI*: 1.4–1.6%). However, the prevalence of adverse events reported in large population (population size > 100 000) was much lower than that in small to medium population size. On the one hand, the vaccination population in the small to medium scale studies we included were mostly composed by health care workers, patients with specific diseases or the elderly. And these people are more concerned about their health and more sensitive to changes of themselves. But it remains to be proved whether patients or the elderly are more likely to have adverse events than the general. Mainstream vaccines currently on the market have maintained robust safety in specific populations such as cancer patients, organ transplant recipients, patients with rheumatic and musculoskeletal diseases, pregnant women and the elderly [54, 91–94]. A prospective study by Tal Goshen-lag suggests that the safety of BNT162b2 vaccine in cancer patients is consistent with those previous reports [91]. In addition, the incidence rate of adverse events reported in the heart–lung transplant population is even lower than that in general population [95]. On the other hand, large scale studies at the national level are mostly based on national electronic health records or adverse event reporting systems, and it is likely that most mild or moderate symptoms are actually not reported.

Compared with the usual local adverse events (such as pain at the injection site, redness at the injection site, etc.) and normal systemic reactions (such as fatigue, myalgia, etc.), serious and life-threatening adverse events were rare due to our results. A meta-analysis based on RCTs only showed three cases of anaphylactic shock among 58 889 COVID-19 vaccine recipients and one in the placebo group [11]. The exact mechanisms underlying most of the adverse events are still unclear, accordingly we cannot establish a causal relation between severe adverse events and vaccination directly based on observational studies. In general, varying degrees of adverse events occur after different types of COVID-19 vaccination. Nevertheless, the benefits far outweigh the risks.

Our results showed the effectiveness and safety of different types of vaccines varied greatly. Regardless of SARS-CoV-2 variants, vaccine effectiveness varied from 66% (CoronaVac [14]) to 97% (mRNA-1273 [18, 20, 45, 46]). The incidence rate of adverse events varied widely among different types of vaccines, which, however, could be explained by the sample size and population group of participants. BNT162b2, AZD1222, mRNA-1273 and CoronaVac were all found to have high vaccine efficacy and acceptable adverse-event profile in recent published studies [96–99]. A meta-analysis, focusing on the potential vaccine candidate which have reached to the phase 3 of clinical development, also found that although many of the vaccines caused more adverse events than the controls, most were mild, transient and manageable [100]. However, severe adverse events did occur, and there remains the need to implement a unified global surveillance system to monitor the adverse events of COVID-19 vaccines around the world [101]. A recent study employed a knowledge-based or rational strategy to perform a prioritization matrix of approved COVID-19 vaccines, and led to a scale with JANSSEN (Ad26.COV2.S) in the first place, and AZD1222, BNT162b2, and Sputnik V in second place, followed by BBIBP-CorV, CoronaVac and mRNA-1273 in third place [101]. Moreover, when deciding the priority of vaccines, the socioeconomic characteristics of each country should also be considered.

Our meta-analysis still has several limitations. First, we may include limited basic data on specific populations, as vaccination is slowly being promoted in populations under the age of 18 or over 60. Second, due to the limitation of the original real-world study, we did not conduct subgroup analysis based on more population characteristics, such as age. When analyzing the efficacy and safety of COVID-19 vaccine, we may have neglected the discussion on the heterogeneity from these sources. Third, most of the original studies only collected adverse events within 7 days after vaccination, which may limit the duration of follow-up for safety analysis.

## Conclusions

Based on the real-world studies, SARS-CoV-2 vaccines have reassuring safety and could effectively reduce the death, severe cases, symptomatic cases, and infections resulting from SARS-CoV-2 across the world. In the context of global pandemic and the continuous emergence of SARS-CoV-2 variants, accelerating vaccination and improving vaccination coverage is still the most important and urgent matter, and it is also the final means to end the pandemic.

## Supplementary Information

**Additional file 1: Table S1.** Characteristic of studies included for vaccine effectiveness.

**Additional file 2: Table S2.** Characteristic of studies included for vaccine safety.

## Data Availability

All data generated or analyzed during this study are included in this published article and its additional information files.

## Abbreviations

COVID-19: Coronavirus disease 2019
SARS-CoV-2: Severe Acute Respiratory Syndrome Coronavirus 2
VE: Vaccine effectiveness
CI: Confidence intervals
ICU: Intensive care unit
RCT: Random clinical trials
PRISMA: Preferred reporting items for systematic reviews and meta-analyses
