# Supplementary material for: Effectiveness and safety of SARS-CoV-2 vaccine in real-world studies: a systematic review and meta-analysis
Source: Infect Dis Poverty. 2021 Nov 14;10:132. doi: 10.1186/s40249-021-00915-3 (PMC8590867; doi:10.1186/s40249-021-00915-3)
Supplement: Supplementary file 1 — Additional file 1: Table S1. Characteristic of studies included for vaccine effectiveness. [file 40249_2021_915_MOESM1_ESM.pdf]

# Table S1. Characteristic of studies included for vaccine effectiveness

| First author | Published time | Study design | Location | Study group | Vaccine type | Vaccination status | Variants of SARS-CoV-2 | Vaccinated people with SARS-CoV-2 infection (n/N) | Unvaccinated people with SARS-CoV-2 infection (n/N) | Vaccine effectiveness (%) | Vaccinated people with symptematic COVID-19 (n/N) | Unvaccinated people with symptematic COVID-19 (n/N) | Vaccine effectiveness (%) | Vaccinated people with COVID-19 hospital admissions (n/N) | Unvaccinated people with COVID-19 hospital admissions (n/N) | Vaccine effectiveness (%) | Vaccinated people with COVID-19 ICU admissions (n/N) | Unvaccinated people with COVID-19 ICU admissions (n/N) | Vaccine effectiveness (%) | Vaccinated people with COVID-19-related death (n/N) | Unvaccinated people with COVID-19-related death (n/N) | Vaccine effectiveness (%) | risk of bias |
|---|---|---|---|---|---|---|---|---|---|---|---|---|---|---|---|---|---|---|---|---|---|---|---|
| Laith J. Abu-Raddad | 2021.7 | case control | Qatar | general population | BNT162b2 | 1 | Alpha (B.1.1.7) | 892/2133 | 18075/35801 | 30 (23, 36) | 30/91 | 468/905 | 54 (26, 72) | .. | .. | .. | .. | .. | .. | .. | .. | .. | low |
| | | | | | | | Beta (B.1.351) | 1329/2909 | 20177/40103 | 17 (10, 23) | 45/80 | 348/706 | 0 (0, 19) | .. | .. | .. | .. | .. | .. | .. | .. | .. | |
| | | | | | | | any | .. | .. | .. | 139/359 | 1966/3851 | 39 (24, 52) | .. | .. | .. | .. | .. | .. | .. | .. | .. | |
| | | | | | | 2(⩾14) | Alpha (B.1.1.7) | 50/515 | 16354/32293 | 90 (86, 92) | 0/20 | 401/782 | 100 (82, 100) | .. | .. | .. | .. | .. | .. | .. | .. | .. | |
| | | | | | | | Beta (B.1.351) | 179/877 | 19396/38273 | 75 (71, 79) | 0/14 | 300/586 | 97 (92, 100) | .. | .. | .. | .. | .. | .. | .. | .. | .. | |
| | | | | | | | any | .. | .. | .. | 3/112 | 1692/3278 | 97 (92, 100) | .. | .. | .. | .. | .. | .. | .. | .. | .. | |
| Yoel Angel* | 2021.5 | cohort study | Israel | Health Care Workers | BNT162b2 | 1(7-28) | | 37/117389 | 8/15091 | 36 (-51, 69) | 31/17420 | 37/15128 | 89 (83, 94) | .. | .. | .. | .. | .. | .. | .. | .. | .. | low |
| | | | | | | 2(⩾7) | | 19/168571 | 17/25359 | 86 (69, 93) | 8/168571 | 38/25359 | 97 (94, 99) | .. | .. | .. | .. | .. | .. | .. | .. | .. | |
| | | | | | | 2(⩾21) | | 4/95689 | 11/15726 | 94 (78, 98) | 2/95691 | 23/15749 | 99 (94, 100) | .. | .. | .. | .. | .. | .. | .. | .. | .. | |
| Tariq Azamgarhi | 2021.6 | cohort study | UK | Health Care Workers | BNT162b2 | 1(<14) | | 16/1408 | 13/825 | 20 (-147, 69) | .. | .. | .. | .. | .. | .. | .. | .. | .. | .. | .. | .. | low |
| | | | | | BNT162b2 | 1(⩾14) | | 7/1392 | 13/812 | 70 (6, 91) | .. | .. | .. | .. | .. | .. | .. | .. | .. | .. | .. | .. | |
| Zacay, G.* | 2021.7 | cohort study | Israel | general | BNT162b2 | 1(⩾14) | | 21/100000 | 53/100000 | 61 (49, 71) | .. | .. | .. | .. | .. | .. | .. | .. | .. | .. | .. | .. | low |
| | | | | | | 2(0-6) | | 9/100000 | 53/100000 | 82 (71, 89) | .. | .. | .. | .. | .. | .. | .. | .. | .. | .. | .. | .. | |
| | | | | | | 2(⩾7) | | 6/100000 | 53/100000 | 89 (82, 94) | .. | .. | .. | .. | .. | .. | .. | .. | .. | .. | .. | .. | |
| Williams, C. | 2021.7 | cohort study | Canada | staff in long term care | BNT162b2 | 2(⩾14) | Gamma (P.1) | 4/43 | 11/40 | 62 (2, 88) | 4/43 | 5/40 | 26 (-158, 79) | .. | .. | .. | .. | .. | .. | .. | .. | .. | moderate |
| | | | | elder residents in long term care | mRNA-1273 | 2(⩾14) | Gamma (P.1) | 19/48 | 10/12 | 53 (27, 70) | 11/48 | 8/12 | 66 (34, 82) | .. | .. | .. | 6/47 | 7/12 | 79 (48, 91) | .. | .. | .. | |
| Francesco Paolo Bianchi | 2021.5 | cohort study | Italy | Health Care Workers | BNT162b2 | 1(14-20) | | .. | .. | 62 (19, 82) | .. | .. | .. | .. | .. | .. | .. | .. | .. | .. | .. | .. | moderate |
| | | | | | | 1(21-27) | | .. | .. | 88 (52, 97) | .. | .. | .. | .. | .. | .. | .. | .. | .. | .. | .. | .. | |
| | | | | | | 2(⩾7) | | .. | .. | 96 (82, 99) | .. | .. | .. | .. | .. | .. | .. | .. | .. | .. | .. | .. | |
| Amadea Britton* | 2021.3 | cohort study | USA | nursing home residents | BNT162b2 | 1(⩾14) | | 25/4172 | 39/3612 | 63 (33, 79) | .. | .. | .. | .. | .. | .. | .. | .. | .. | .. | .. | .. | moderate |
| Martínez-Baz, I. | 2021.5 | cohort study | Spain | adults who had been close contacts of laboratory-confirmed COVID-19 cases from January to April 2021. | Mix | 1(⩾14) | | 198/869 | 6980/19580 | 35 (25, 44) | 135/869 | 5306/19580 | 42 (31, 52) | 10/869 | 548/19580 | 72 (47, 85) | .. | .. | .. | .. | .. | .. | low |
| | | | | | | 2(⩾14) | | 62/512 | 6980/19580 | 66 (57, 74) | 26/512 | 5306/19580 | 82 (74, 88) | 1/512 | 548/19580 | 95 (62, 99) | .. | .. | .. | .. | .. | .. | |
| | | | | | AZD1222 | 1(⩾14) | | 99/524 | 6980/19580 | .. | 67/524 | 5306/19580 | .. | 1/524 | 548/19580 | .. | .. | .. | .. | .. | .. | .. | |
| | | | | | BNT162b2 | 2(⩾14) | | 61/492 | 6980/19580 | .. | 25/491 | 5306/19580 | .. | 1/491 | 548/19580 | .. | .. | .. | .. | .. | .. | .. | |
| | | | | | mRNA-1273 | 1(⩾14) | | 90/310 | 6980/19580 | .. | 62/310 | 5306/19580 | .. | 7/310 | 548/19580 | .. | .. | .. | .. | .. | .. | .. | |
| | | | | | | 1(⩾14) | | 9/35 | 6980/19580 | .. | 6/35 | 5306/19580 | .. | 2/35 | 548/19580 | .. | .. | .. | .. | .. | .. | .. | |
| | | | | | | 2(⩾14) | | 1月21日 | 6980/19580 | .. | 1/21 | 5306/19580 | .. | 0/21 | 548/19580 | .. | .. | .. | .. | .. | .. | .. | |
| Swift, M. D. | 2021.4 | cohort study | USA | healthcare personnel | BNT162b2 | 1(>14) and 2(0-14) | | .. | .. | 78 (77, 82) | .. | .. | .. | .. | .. | .. | .. | .. | .. | .. | .. | .. | low |
| | | | | | | 2(>14) | | .. | .. | 97 (95, 98) | .. | .. | .. | .. | .. | .. | .. | .. | .. | .. | .. | .. | |
| | | | | | mRNA-1273 | 2(>14) | | .. | .. | 99 (90, 100) | .. | .. | .. | .. | .. | .. | .. | .. | .. | .. | .. | .. | |
| | | | | | | 1(>14) and 2(0-14) | | .. | .. | 91 (81, 96) | .. | .. | .. | .. | .. | .. | .. | .. | .. | .. | .. | .. | |
| | | | | | mRNA-1273+BNT162b2 | 1(>14) and 2(0-14) | | 98/3204 | 997/23931 | .. | 66/3204 | 876/23931 | .. | .. | .. | .. | .. | .. | .. | .. | .. | .. | |
| | | | | | | 2(>14) | | 30/44002 | 997/23931 | .. | 22/44002 | 876/23931 | .. | .. | .. | .. | .. | .. | .. | .. | .. | .. | |
| Mazagatos, C. | 2021.6 | case-control | Spain | elderly long-term care facility residents | mRNA-1273+BNT162b2 | 1(⩾14) | | 1559/.. | 6820/.. | 51 (37, 61) | .. | .. | .. | 404/.. | 2105/.. | 53 (26, 70) | .. | .. | .. | .. | .. | 56 (27, 73) | low |
| | | | | | | 2(0-6 for Comirnaty or 0-14 for Moderna) | | 413/.. | 7966/.. | 79 (67, 86) | 101/.. | 2408/.. | 83 (61, 93) | .. | .. | .. | .. | .. | .. | .. | .. | 96 (83, 99) | |
| | | | | | | 2(⩾7 for Comirnaty or ⩾14 for Moderna) | | 292/300425 | 8087/46099 | 71 (56, 82) | 49/300182 | 160/38172 | 88 (75, 95) | .. | .. | .. | .. | .. | .. | .. | .. | 97 (92, 99) | |
| Alyson M. Cavanaugh | 2021.4 | cross-sectional | USA | nursing home residents | BNT162b2 | 2(⩾14) | | 18/71 | 6/8 | 66 (41, 81) | 6/71 | 5/8 | 87 (66, 95) | 2/71 | 4/8 | 94 (74, 99) | .. | .. | .. | 1/71 | 2/8 | 94 (45, 99) | moderate |
| | | | | Health Care Workers | BNT162b2 | 2(⩾14) | | 4/56 | 16/54 | 76 (33, 91) | 2/56 | 15/54 | 87 (46, 97) | .. | .. | .. | .. | .. | .. | .. | .. | .. | |
| Tenforde, M. W. | 2021.5 | case-control | USA | Hospitalized Adults Aged ⩾65 Years | mRNA-1273+BNT162b2 | 1(0-13) | | 22/49 | 146/287 | 3 (-94, 51) | .. | .. | .. | .. | .. | .. | .. | .. | .. | .. | .. | .. | moderate |
| | | | | | | 1(⩾14) and 2(0-13) | | 18/62 | 146/287 | 64 (28, 82) | .. | .. | .. | .. | .. | .. | .. | .. | .. | .. | .. | .. | |
| | | | | | | 2(⩾14) | | 1/19 | 146/287 | 94 (49, 99) | .. | .. | .. | .. | .. | .. | .. | .. | .. | .. | .. | .. | |
| Hiam Chemaitelly | 2021.7 | case control | Qatar | general population | mRNA-1273 | 1(<7) | Alpha (B.1.1.7) | 166/336 | 23661/47318 | 2 (0, 22) | .. | .. | .. | .. | .. | .. | .. | .. | .. | .. | .. | .. | low |
| | | | | | | | Beta (B.1.351) | 535/1093 | 47359/94695 | 4 (0, 15) | .. | .. | .. | .. | .. | .. | .. | .. | .. | .. | .. | .. | |
| | | | | | | | any | .. | .. | .. | 53/118 | 4012/8012 | 19 (0, 45) | .. | .. | .. | .. | .. | .. | .. | .. | .. | |
| | | | | | | 1(7-13) | Alpha (B.1.1.7) | 202/389 | 23681/47377 | 0 (0, 12) | .. | .. | .. | .. | .. | .. | .. | .. | .. | .. | .. | .. | |
| | | | | | | | Beta (B.1.351) | 854/1420 | 47372/95032 | 0 (0, 0) | .. | .. | .. | .. | .. | .. | .. | .. | .. | .. | .. | .. | |
| | | | | | | | any | .. | .. | .. | 85/153 | 4016/8049 | 0 (0, 10) | .. | .. | .. | .. | .. | .. | .. | .. | .. | |
| | | | | | | 1(14-20) | Alpha (B.1.1.7) | 32/205 | 23669/47197 | 82 (73, 88) | .. | .. | .. | .. | .. | .. | .. | .. | .. | .. | .. | .. | |
| | | | | | | | Beta (B.1.351) | 270/786 | 47329/94412 | 48 (40, 55) | .. | .. | .. | .. | .. | .. | .. | .. | .. | .. | .. | .. | |
| | | | | | | | any | .. | .. | .. | 18/78 | 4007/7972 | 70 (49, 84) | .. | .. | .. | .. | .. | .. | .. | .. | .. | |
| | | | | | | 1(21-27) | Alpha (B.1.1.7) | 9/169 | 23656/47161 | 94 (89, 98) | .. | .. | .. | .. | .. | .. | .. | .. | .. | .. | .. | .. | |
| | | | | | | | Beta (B.1.351) | 114/545 | 47249/94181 | 74 (68, 79) | .. | .. | .. | .. | .. | .. | .. | .. | .. | .. | .. | .. | |
| | | | | | | | any | .. | .. | .. | 4/54 | 3997/7948 | 92 (78, 98) | .. | .. | .. | .. | .. | .. | .. | .. | .. | |
| | | | | | | 2(<7) | Alpha (B.1.1.7) | 4/200 | 21454/42716 | 98 (95, 100) | .. | .. | .. | .. | .. | .. | .. | .. | .. | .. | .. | .. | |

| | | | | | | | | | | | | | | | | | | | | | | | |
|---|---|---|---|---|---|---|---|---|---|---|---|---|---|---|---|---|---|---|---|---|---|---|---|
| | | | | | | | Beta (B.1.351) | 42/761 | 45280/89883 | 94 (92, 96) | .. | .. | .. | .. | .. | .. | .. | .. | .. | .. | .. | .. | |
| | | | | | | | any | .. | .. | .. | 0/62 | 3432/6802 | 100 (94, 100) | .. | .. | .. | .. | .. | .. | .. | .. | .. | |
| | | | | | | 2(7-13) | Alpha (B.1.1.7) | 1/120 | 21377/42636 | 99 (95, 100) | .. | .. | .. | .. | .. | .. | .. | .. | .. | .. | .. | .. | |
| | | | | | | | Beta (B.1.351) | 18/516 | 45064/89648 | 96 (94, 98) | .. | .. | .. | .. | .. | .. | .. | .. | .. | .. | .. | .. | |
| | | | | | | | any | .. | .. | .. | 0/29 | 3399/6769 | 100 (87, 100) | .. | .. | .. | .. | .. | .. | .. | .. | .. | |
| Pawlowski, C.* | 2021.7 | cohort study | USA | general population | BNT162b2 | 1(≥7) | | 401/2942986 | 1232/2851069 | 69 (65, 72) | .. | .. | .. | .. | .. | .. | .. | .. | .. | .. | .. | .. | low |
| | | | | | | 1(≥14) | | 211/2595549 | 960/2506231 | 79 (75, 82) | .. | .. | .. | .. | .. | .. | .. | .. | .. | .. | .. | .. | |
| | | | | | | 1(≥7 before 2) | | 293/766978 | 534/761511 | 46 (37, 53) | .. | .. | .. | .. | .. | .. | .. | .. | .. | .. | .. | .. | |
| | | | | | | 1(≥14 before 2) | | 103/419541 | 262/416673 | 61 (51, 69) | .. | .. | .. | .. | .. | .. | .. | .. | .. | .. | .. | .. | |
| | | | | | | 2(≥7) | | 82/1914500 | 563/1828466 | 86 (82, 89) | .. | .. | .. | 7/1915615 | 60/1837267 | 89 (76, 96) | 0/1915733 | 9/1838072 | 100 (51, 100) | .. | .. | .. | |
| | | | | | | 2(≥14) | | 59/1670896 | 468/1591900 | 88 (84, 91) | .. | .. | .. | 6/1671628 | 49/1599076 | 88 (73, 96) | 0/1671730 | 6/1599732 | 100 (19, 100) | .. | .. | .. | |
| | | | | | mRNA-1273 | 1(≥7) | | 97/946890 | 303/927716 | 69 (61, 75) | .. | .. | .. | .. | .. | .. | .. | .. | .. | .. | .. | .. | |
| | | | | | | 1(≥14) | | 49/833098 | 241/814413 | 80 (73, 86) | .. | .. | .. | .. | .. | .. | .. | .. | .. | .. | .. | .. | |
| | | | | | | 1(≥7 before 2) | | 88/369195 | 181/366881 | 52 (37, 63) | .. | .. | .. | .. | .. | .. | .. | .. | .. | .. | .. | .. | |
| | | | | | | 1(≥14 before 2) | | 40/255403 | 119/253578 | 67 (52, 77) | .. | .. | .. | .. | .. | .. | .. | .. | .. | .. | .. | .. | |
| | | | | | | 2(≥7) | | 7/495550 | 101/478322 | 93 (86, 97) | .. | .. | .. | 9/948311 | 63/932315 | 86 (72, 94) | 0/495630 | 8/479979 | 100 (43, 100) | .. | .. | .. | |
| | | | | | | 2(≥14) | | 6/417664 | 75/402416 | 92 (82, 97) | .. | .. | .. | 5/833681 | 52/817970 | 91 (76, 97) | 0/417743 | 6/403589 | 100 (18, 100) | .. | .. | .. | |
| Gabriel Chodick | 2021.6 | cohort study | Israel | general population | BNT162b2 | 1(13-24) | | 614/.. | 2484/.. | 51 (16, 72) | 406/.. | 1692/.. | 54 (21, 74) | .. | .. | .. | .. | .. | .. | .. | .. | .. | low |
| Gabriel Chodick | 2021.5 | cohort study | Israel | general population | BNT162b2 | 2(7-27) | | 728/27456 | 4514/60913 | 90 (79, 95) | .. | .. | .. | .. | .. | .. | .. | .. | .. | .. | .. | .. | low |
| Pilishvili, T. | 2021.5 | case control | USA | Health Care Personnel | mRNA-1273+BNT162b2 | 1(>14) and 2(0-6) | | 64/305 | 559/1538 | 82 (74, 87) | .. | .. | .. | .. | .. | .. | .. | .. | .. | .. | .. | .. | low |
| | | | | | | 2(≥7) | | 40/418 | 583/1425 | 94 (87, 97) | .. | .. | .. | .. | .. | .. | .. | .. | .. | .. | .. | .. | |
| Vasileiou, E. | 2021.4 | cohort study | Scotland | | BNT162b2 | 1(0–6) | | .. | .. | .. | .. | .. | .. | 156/19917 | 7698/831226 | 75 (71, 79) | .. | .. | .. | .. | .. | .. | low |
| | | | | | | 1(7–13) | | .. | .. | .. | .. | .. | .. | 258/19690 | 7698/831226 | 54 (48, 59) | .. | .. | .. | .. | .. | .. | |
| | | | | | | 1(14–20) | | .. | .. | .. | .. | .. | .. | 154/13426 | 7698/831226 | 67 (61, 72) | .. | .. | .. | .. | .. | .. | |
| | | | | | | 1(21–27) | | .. | .. | .. | .. | .. | .. | 72/9119 | 7698/831226 | 76 (70, 81) | .. | .. | .. | .. | .. | .. | |
| | | | | | | 1(28–34) | | .. | .. | .. | .. | .. | .. | 30/6252 | 7698/831226 | 89 (83, 92) | .. | .. | .. | .. | .. | .. | |
| | | | | | | 1(35–41) | | .. | .. | .. | .. | .. | .. | 21/3822 | 7698/831226 | 78 (68, 85) | .. | .. | .. | .. | .. | .. | |
| | | | | | | 1(≥42) | | .. | .. | .. | .. | .. | .. | 32/6047 | 7698/831226 | 74 (65, 81) | .. | .. | .. | .. | .. | .. | |
| | | | | | AZD1222 | 1(0–6) | | .. | .. | .. | .. | .. | .. | 122/9222 | 7252/743142 | 72 (66, 77) | .. | .. | .. | .. | .. | .. | |
| | | | | | | 1(7–13) | | .. | .. | .. | .. | .. | .. | 139/8699 | 7252/743142 | 68 (61, 73) | .. | .. | .. | .. | .. | .. | |
| | | | | | | 1(14–20) | | .. | .. | .. | .. | .. | .. | 83/5742 | 7252/743142 | 73 (66, 79) | .. | .. | .. | .. | .. | .. | |
| | | | | | | 1(21–27) | | .. | .. | .. | .. | .. | .. | 34/2447 | 7252/743142 | 81 (72, 87) | .. | .. | .. | .. | .. | .. | |
| | | | | | | 1(28–34) | | .. | .. | .. | .. | .. | .. | 11/1666 | 7252/743142 | 88 (75, 94) | .. | .. | .. | .. | .. | .. | |
| | | | | | | 1(35–41) | | .. | .. | .. | .. | .. | .. | ≤5/530 | 7252/743142 | 97 (63, 100) | .. | .. | .. | .. | .. | .. | |
| | | | | | | 1(≥42) | | .. | .. | .. | .. | .. | .. | ≤5/51 | 7252/743142 | 59 (-296, 96) | .. | .. | .. | .. | .. | .. | |
| Noa Dagan | 2021.2 | cohort study | Israel | general population | BNT162b2 | 1(14-20) | | .. | .. | 46 (40, 51) | .. | .. | 57 (50, 63) | .. | .. | 74 (56, 86) | .. | .. | 62 (39, 80) | .. | .. | .. | low |
| | | | | | | 2(≥7) | | .. | .. | 92 (88, 95) | .. | .. | 94 (87, 98) | .. | .. | 87 (55, 100) | .. | .. | 92 (75, 100) | .. | .. | .. | |
| Maria Elena Flacco | 2021.6 | cohort study | Italy | general population | BNT162b2 | 1 | | .. | .. | 55 (40, 66) | .. | .. | .. | .. | .. | .. | .. | .. | .. | .. | .. | .. | low |
| | | | | | | 2 | | .. | .. | 98 (96, 99) | .. | .. | 99 (96, 100) | .. | .. | .. | .. | .. | .. | .. | .. | 98 (87, 100) | |
| | | | | | | ≥1 | | .. | .. | 95 (93, 96) | .. | .. | 95 (92, 97) | .. | .. | .. | .. | .. | .. | .. | .. | 97 (91, 99) | |
| | | | | | mRNA-1273 | 1 | | .. | .. | 93 (74, 98) | .. | .. | .. | .. | .. | .. | .. | .. | .. | .. | .. | .. | |
| Thompson, M. G. | 2021.7 | cohort study | USA | health care personnel, first responders, and other essential and frontline workers | BNT162b2 | 1(≥14) and 2(0-13) | | 8/2005 | 156/3964 | 80 (60, 90) | .. | .. | .. | .. | .. | .. | .. | .. | .. | .. | .. | .. | low |
| | | | | | | 2(≥14) | | 3/1731 | 156/3964 | 93 (78, 98) | .. | .. | .. | .. | .. | .. | .. | .. | .. | .. | .. | .. | |
| | | | | | mRNA-1273 | 1(≥14) and 2(0-13) | | 3/982 | 156/3964 | 83 (40, 95) | .. | .. | .. | .. | .. | .. | .. | .. | .. | .. | .. | .. | |
| | | | | | | 2(≥14) | | 2/770 | 156/3964 | 82 (20, 96) | .. | .. | .. | .. | .. | .. | .. | .. | .. | .. | .. | .. | |
| Eric J Haas | 2021.5 | cohort study | Israel | general population | BNT162b2 | 2(≥7) | | 3·1/100000 | 91·5/100000 | 95 (95, 96) | 0·8/100000 | 32·5/100000 | 97 (97, 97) | 0·3/100000 | 4·6/100000 | 97 (97, 98) | 0·2/100000 | 2·7/100000 | 98 (97, 98) | 0·1/100000 | 0·6/100000 | 97 (96, 97) | low |
| | | | | | | 2(≥14) | | 2·1/100000 | 91·5/100000 | 97 (96, 97) | 0·6/100000 | 32·5/100000 | 98 (98, 98) | 0·2/100000 | 4·6/100000 | 98 (98, 98) | 0·1/100000 | 2·7/100000 | 98 (98, 99) | <0·1/100000 | 0·6/100000 | 98 (98, 98) | |
| Victoria Jane Hall* | 2021.5 | cohort study | UK | Health Care Workers | BNT162b2+AZD1222 | 1(>21) | | 71/87278 | 977/710587 | 70 (55, 85) | .. | .. | .. | .. | .. | .. | .. | .. | .. | .. | .. | .. | low |
| | | | | | | 2(≥7) | | 9/20978 | 977/710587 | 85 (74, 96) | .. | .. | .. | .. | .. | .. | .. | .. | .. | .. | .. | .. | |
| Catherine Hyams | 2021.6 | case control | UK | people aged at least 80 years | AZD1222 | 1 | | .. | .. | .. | 9/62 | 27/64 | 80 (36, 95) | .. | .. | .. | .. | .. | .. | .. | .. | .. | low |
| | | | | | BNT162b2 | 1 | | .. | .. | .. | 18/108 | 117/296 | 71 (43, 86) | .. | .. | .. | .. | .. | .. | .. | .. | .. | |
| Sheikh, A. | 2021.6 | cohort study | Scotland | | BNT162b2 | 1(0-27) | Alpha (B.1.1.7) | 188/6857 | 5828/119419 | 31 (20, 41) | 119/4116 | 3997/42062 | 28 (13, 41) | .. | .. | .. | .. | .. | .. | .. | .. | .. | low |
| | | | | | | | Delta (B.1.617.2) | 317/6986 | 3672/117263 | 12 (0, 22) | 203/388 | 2439/40504 | 18 (4, 31) | .. | .. | .. | .. | .. | .. | .. | .. | .. | |
| | | | | | | 1(≥28) | Alpha (B.1.1.7) | 273/14324 | 5828/119419 | 38 (29, 45) | 182/2466 | 3997/42062 | 27 (13, 39) | .. | .. | .. | .. | .. | .. | .. | .. | .. | |
| | | | | | | | Delta (B.1.617.2) | 163/14114 | 3672/117263 | 30 (17, 41) | 92/2376 | 2439/40504 | 33 (15, 47) | .. | .. | .. | .. | .. | .. | .. | .. | .. | |
| | | | | | | 2(0-13) | Alpha (B.1.1.7) | 59/7277 | 3672/117263 | 73 (64, 79) | 20/898 | 3997/42062 | 78 (65, 86) | .. | .. | .. | .. | .. | .. | .. | .. | .. | |
| | | | | | | | Delta (B.1.617.2) | 15/7233 | 3672/117263 | 66 (43, 80) | 5/883 | 2439/40504 | 84 (61, 93) | .. | .. | .. | .. | .. | .. | .. | .. | .. | |
| | | | | | | 2(≥14) | Alpha (B.1.1.7) | 104/53575 | 3672/117263 | 92 (90, 93) | 34/4360 | 3997/42062 | 92 (88, 94) | .. | .. | .. | .. | .. | .. | .. | .. | .. | |
| | | | | | | | Delta (B.1.617.2) | 208/53679 | 3672/117263 | 79 (75, 82) | 75/4401 | 2439/40504 | 83 (78, 87) | .. | .. | .. | .. | .. | .. | .. | .. | .. | |
| | | | | | AZD1222 | 1(0-27) | Alpha (B.1.1.7) | 567/15137 | 5828/119419 | 9 (-1, 17) | 398/4634 | 3997/42062 | 17 (6, 26) | .. | .. | .. | .. | .. | .. | .. | .. | .. | |
| | | | | | | | Delta (B.1.617.2) | 293/14863 | 3672/117263 | 7 (-7, 19) | 186/4422 | 2439/40504 | 23 (7, 36) | .. | .. | .. | .. | .. | .. | .. | .. | .. | |
| | | | | | | 1(≥28) | Alpha (B.1.1.7) | 956/51572 | 5828/119419 | 37 (32, 42) | 591/10322 | 3997/42062 | 39 (32, 45) | .. | .. | .. | .. | .. | .. | .. | .. | .. | |
| | | | | | | | Delta (B.1.617.2) | 776/51392 | 3672/117263 | 18 (9, 25) | 511/10242 | 2439/40504 | 33 (23, 41) | .. | .. | .. | .. | .. | .. | .. | .. | .. | |
| | | | | | | 2(0-13) | Alpha (B.1.1.7) | 99/13818 | 3672/117263 | 64 (56, 71) | 57/1774 | 3997/42062 | 65 (54, 74) | .. | .. | .. | .. | .. | .. | .. | .. | .. | |
| | | | | | | | Delta (B.1.617.2) | 265/13975 | 3672/117263 | 25 (14, 35) | 160/1877 | 2439/40504 | 37 (23, 48) | .. | .. | .. | .. | .. | .. | .. | .. | .. | |
| | | | | | | 2(≥14) | Alpha (B.1.1.7) | 100/32588 | 3672/117263 | 73 (66, 78) | 36/1999 | 3997/42062 | 81 (72, 87) | .. | .. | .. | .. | .. | .. | .. | .. | .. | |
| | | | | | | | Delta (B.1.617.2) | 231/32719 | 3672/117263 | 60 (53, 66) | 126/2089 | 2439/40504 | 61 (51, 70) | .. | .. | .. | .. | .. | .. | .. | .. | .. | |
| Alejandro Jara* | 2021.7 | cohort study | Chile | general population | CoronaVac | 1(14-28) | | 20865/69788352 | 185633/614868240 | 16 (14, 17) | .. | .. | .. | 3370/70690796 | 18034/620894706 | 37 (35, 40) | 1154/70836597 | 6359/621226431 | 45 (41, 48) | 847/70854187 | 2786/621426477 | 46 (41, 50) | low |

| | | | | | | | | | | | | | | | | | | | | | | | |
|---|---|---|---|---|---|---|---|---|---|---|---|---|---|---|---|---|---|---|---|---|---|---|---|
| | | | | | | 2(≥14) | | 12286/91671797 | 185633/614868240 | 66 (65, 67) | .. | .. | .. | 1462/92445333 | 18034/620894706 | 88 (87, 88) | 360/92622083 | 6359/621226431 | 90 (89, 91) | 409/92514261 | 2786/621426477 | 86 (84, 88) | |
| Nabeel Khan | 2021.5 | cohort study | USA | Patients With Inflammatory Bowel Disease With Diverse Exposure to Immunosuppressive Medications | mRNA-1273+BNT162b2 | 1(≥14) | | .. | .. | -1 (-50, 32) | .. | .. | .. | .. | .. | .. | .. | .. | 9 (-114, 61) | .. | .. | -27 (-116, 25) | low |
| | | | | | | 2(≥7) | | .. | .. | 69 (44, 83) | .. | .. | .. | .. | .. | .. | .. | .. | 49 (-36, 81) | .. | .. | 57 (-19, 85) | |
| Skowronski, D. M. | 2021.7 | case control | British Columbia, Canada | adults 70 years and older | Not mentioned | 1(0-13) | Alpha (B.1.1.7) | 140/3218 | 200/4267 | 18 (-3, 34) | .. | .. | .. | .. | .. | .. | .. | .. | .. | .. | .. | .. | low |
| | | | | | | | Gamma (P.1) | 81/3159 | 109/4176 | 11 (-19, 34) | .. | .. | .. | .. | .. | .. | .. | .. | .. | .. | .. | .. | |
| | | | | | | | any | 81/3159 | 109/4176 | 17 (-10, 38) | .. | .. | .. | .. | .. | .. | .. | .. | .. | .. | .. | .. | |
| | | | | | | 1(14-20) | Alpha (B.1.1.7) | 66/2097 | 200/4267 | 48 (30, 61) | .. | .. | .. | .. | .. | .. | .. | .. | .. | .. | .. | .. | |
| | | | | | | | Gamma (P.1) | 53/2084 | 109/4176 | 24 (-19, 34) | .. | .. | .. | .. | .. | .. | .. | .. | .. | .. | .. | .. | |
| | | | | | | | any | 24/2055 | 126/4193 | 63 (43, 77) | .. | .. | .. | .. | .. | .. | .. | .. | .. | .. | .. | .. | |
| | | | | | | 1(21-27) | Alpha (B.1.1.7) | 52/2244 | 200/4267 | 55 (37, 67) | .. | .. | .. | .. | .. | .. | .. | .. | .. | .. | .. | .. | |
| | | | | | | | Gamma (P.1) | 30/2222 | 109/4176 | 52 (26, 69) | .. | .. | .. | .. | .. | .. | .. | .. | .. | .. | .. | .. | |
| | | | | | | | any | 15/2207 | 126/4193 | 74 (55, 85) | .. | .. | .. | .. | .. | .. | .. | .. | .. | .. | .. | .. | |
| | | | | | | 1(28-34) | Alpha (B.1.1.7) | 22/1812 | 200/4267 | 76 (62, 85) | .. | .. | .. | .. | .. | .. | .. | .. | .. | .. | .. | .. | |
| | | | | | | | Gamma (P.1) | 18/1808 | 109/4176 | 66 (42, 80) | .. | .. | .. | .. | .. | .. | .. | .. | .. | .. | .. | .. | |
| | | | | | | | any | 12/1802 | 126/4193 | 70 (43, 84) | .. | .. | .. | .. | .. | .. | .. | .. | .. | .. | .. | .. | |
| | | | | | | 1(35-41) | Alpha (B.1.1.7) | 11/1191 | 200/4267 | 81 (64, 90) | .. | .. | .. | .. | .. | .. | .. | .. | .. | .. | .. | .. | |
| | | | | | | | Gamma (P.1) | 9/1189 | 109/4176 | 68 (35, 85) | .. | .. | .. | .. | .. | .. | .. | .. | .. | .. | .. | .. | |
| | | | | | | | any | 9/1189 | 126/4193 | 65 (27, 83) | .. | .. | .. | .. | .. | .. | .. | .. | .. | .. | .. | .. | |
| | | | | | | 1(≥21) | Alpha (B.1.1.7) | 103/6424 | 200/4267 | 67 (57, 75) | .. | .. | .. | .. | .. | .. | .. | .. | .. | .. | .. | .. | |
| | | | | | | | Gamma (P.1) | 71/6392 | 109/4176 | 61 (45, 72) | .. | .. | .. | .. | .. | .. | .. | .. | .. | .. | .. | .. | |
| | | | | | | | any | 42/6363 | 126/4193 | 72 (58, 81) | .. | .. | .. | .. | .. | .. | .. | .. | .. | .. | .. | .. | |
| | | | | | | 1(≥42) | Alpha (B.1.1.7) | 18/1168 | 200/4267 | 67 (44, 81) | .. | .. | .. | .. | .. | .. | .. | .. | .. | .. | .. | .. | |
| | | | | | | | Gamma (P.1) | 14/1164 | 109/4176 | 53 (12, 75) | .. | .. | .. | .. | .. | .. | .. | .. | .. | .. | .. | .. | |
| | | | | | | | any | 6/1156 | 126/4193 | 72 (33, 88) | .. | .. | .. | .. | .. | .. | .. | .. | .. | .. | .. | .. | |
| | | | | | | 1(0-13) | any | .. | .. | 14 (0, 26) | .. | .. | .. | .. | .. | .. | .. | .. | .. | .. | .. | .. | |
| | | | | | | 1(14-20) | any | .. | .. | 43 (30, 53) | .. | .. | .. | .. | .. | .. | .. | .. | .. | .. | .. | .. | |
| | | | | | | 1(35-41) | any | .. | .. | 75 (63, 83) | .. | .. | .. | .. | .. | .. | .. | .. | .. | .. | .. | .. | |
| Pablo Knobel | 2021.6 | cohort study | Spain | Health Care Workers | mRNA-1273+BNT162b2 | 1(<14) | | 32/2076 | 94/6767 | .. | .. | .. | .. | .. | .. | .. | .. | .. | .. | .. | .. | .. | moderate |
| | | | | | | 1(≥14) | | 19/2350 | 94/6767 | .. | .. | .. | .. | .. | .. | .. | .. | .. | .. | .. | .. | .. | |
| | | | | | | 2(≥7) | | 7/5530 | 94/6767 | .. | .. | .. | .. | .. | .. | .. | .. | .. | .. | .. | .. | .. | |
| Jamie Lopez Bernal | 2021.5 | case control | UK | older adults | BNT162b2 | 1(0-3) | | 167/444 | 8988/24706 | -22 (-48, 0) | .. | .. | .. | .. | .. | .. | .. | .. | .. | .. | .. | .. | low |
| | | | | | | 1(4-6) | | 179/420 | 8988/24706 | -28 (-56, -5) | .. | .. | .. | .. | .. | .. | .. | .. | .. | .. | .. | .. | |
| | | | | | | 1(7-9) | | 257/509 | 8988/24706 | -48 (-77, -23) | .. | .. | .. | .. | .. | .. | .. | .. | .. | .. | .. | .. | |
| | | | | | | 1(10-13) | | 284/645 | 8988/24706 | -13 (-33, 4) | .. | .. | .. | .. | .. | .. | .. | .. | .. | .. | .. | .. | |
| | | | | | | 1(14-20) | | 336/798 | 8988/24706 | -6 (-23, 8) | .. | .. | .. | .. | .. | .. | .. | .. | .. | .. | .. | .. | |
| | | | | | | 1(21-27) | | 118/406 | 8988/24706 | 36 (21, 49) | .. | .. | .. | .. | .. | .. | .. | .. | .. | .. | .. | .. | |
| | | | | | | 1(28-30) | | 72/362 | 8988/24706 | 59 (46, 68) | .. | .. | .. | .. | .. | .. | .. | .. | .. | .. | .. | .. | |
| | | | | | | 1(35-41) | | 65/339 | 8988/24706 | 51 (34, 63) | .. | .. | .. | .. | .. | .. | .. | .. | .. | .. | .. | .. | |
| | | | | | | 1(≥42) | | 59/455 | 8988/24706 | 61 (45, 71) | .. | .. | .. | .. | .. | .. | .. | .. | .. | .. | .. | .. | |
| | | | | | | 2(0-3) | | 45/161 | 8988/24706 | 41 (17, 59) | .. | .. | .. | .. | .. | .. | .. | .. | .. | .. | .. | .. | |
| | | | | | | 2(4-6) | | 30/110 | 8988/24706 | 43 (12, 63) | .. | .. | .. | .. | .. | .. | .. | .. | .. | .. | .. | .. | |
| | | | | | | 2(7-13) | | 28/229 | 8988/24706 | 79 (68, 86) | .. | .. | .. | .. | .. | .. | .. | .. | .. | .. | .. | .. | |
| | | | | | | 2(≥14) | | 41/675 | 8988/24706 | 85 (79, 89) | .. | .. | .. | .. | .. | .. | .. | .. | .. | .. | .. | .. | |
| Shrotri, M.* | 2021.6 | cohort study | England | residents of long-term care facilities | BNT162b2 | 1(0-6) | | 11·95/10000 | 21·39/10000 | 16 (-81, 61) | .. | .. | .. | .. | .. | .. | .. | .. | .. | .. | .. | .. | low |
| | | | | | | 1(7-13) | | 8·13/10000 | 21·39/10000 | -11 (-88, 35) | .. | .. | .. | .. | .. | .. | .. | .. | .. | .. | .. | .. | |
| | | | | | | 1(14-20) | | 11·8/10000 | 21·39/10000 | 23 (-58, 63) | .. | .. | .. | .. | .. | .. | .. | .. | .. | .. | .. | .. | |
| | | | | | | 1(21-27) | | 26·67/10000 | 21·39/10000 | 6 (-79, 50) | .. | .. | .. | .. | .. | .. | .. | .. | .. | .. | .. | .. | |
| | | | | | | 1(28-34) | | 28·26/10000 | 21·39/10000 | 53 (-6, 80) | .. | .. | .. | .. | .. | .. | .. | .. | .. | .. | .. | .. | |
| | | | | | | 1(35-48) | | 38·6/10000 | 21·39/10000 | 65 (29, 83) | .. | .. | .. | .. | .. | .. | .. | .. | .. | .. | .. | .. | |
| | | | | | | 1(≥49) | | 26·24/10000 | 21·39/10000 | 62 (7, 85) | .. | .. | .. | .. | .. | .. | .. | .. | .. | .. | .. | .. | |
| | | | | | AZD1222 | 1(0-6) | | 19·79/10000 | 21·39/10000 | 51 (1, 100) | .. | .. | .. | .. | .. | .. | .. | .. | .. | .. | .. | .. | |
| | | | | | | 1(7-13) | | 19·08/10000 | 21·39/10000 | 42 (4, 65) | .. | .. | .. | .. | .. | .. | .. | .. | .. | .. | .. | .. | |
| | | | | | | 1(14-20) | | 25·1/10000 | 21·39/10000 | 5 (-84, 50) | .. | .. | .. | .. | .. | .. | .. | .. | .. | .. | .. | .. | |
| | | | | | | 1(21-27) | | 16·32/10000 | 21·39/10000 | 27 (-44, 63) | .. | .. | .. | .. | .. | .. | .. | .. | .. | .. | .. | .. | |
| | | | | | | 1(28-34) | | 8·51/10000 | 21·39/10000 | 67 (32, 84) | .. | .. | .. | .. | .. | .. | .. | .. | .. | .. | .. | .. | |
| | | | | | | 1(35-48) | | 10·37/10000 | 21·39/10000 | 68 (34, 85) | .. | .. | .. | .. | .. | .. | .. | .. | .. | .. | .. | .. | |
| | | | | | | 1(≥49) | | 21·56/10000 | 21·39/10000 | 36 (-56, 74) | .. | .. | .. | .. | .. | .. | .. | .. | .. | .. | .. | .. | |
| Jamie Lopez Bernal | 2021.7 | case control | UK | general population | AZD1222 | 1(>21) | Alpha (B.1.1.7) | 1776/44605 | 7313/103684 | 49 (45, 52) | .. | .. | .. | .. | .. | .. | .. | .. | .. | .. | .. | .. | |
| | | | | | | | Delta (B.1.617.2) | 1356/44185 | 4043/100414 | 30 (24, 35) | .. | .. | .. | .. | .. | .. | .. | .. | .. | .. | .. | .. | |
| | | | | | | 2(≥14) | Alpha (B.1.1.7) | 94/8338 | 7313/103684 | 75 (68, 79) | .. | .. | .. | .. | .. | .. | .. | .. | .. | .. | .. | .. | |
| | | | | | | | Delta (B.1.617.2) | 218/8462 | 4043/100414 | 67 (61, 72) | .. | .. | .. | .. | .. | .. | .. | .. | .. | .. | .. | .. | |
| | | | | | BNT162b2 | 1(>21) | Alpha (B.1.1.7) | 450/9091 | 7313/103684 | 48 (42, 53) | .. | .. | .. | .. | .. | .. | .. | .. | .. | .. | .. | .. | low |
| | | | | | | | Delta (B.1.617.2) | 137/8778 | 4043/100414 | 36 (23, 46) | .. | .. | .. | .. | .. | .. | .. | .. | .. | .. | .. | .. | |
| | | | | | | 2(≥14) | Alpha (B.1.1.7) | 49/15798 | 7313/103684 | 94 (92, 95) | .. | .. | .. | .. | .. | .. | .. | .. | .. | .. | .. | .. | low |
| | | | | | | | Delta (B.1.617.2) | 122/15871 | 4043/100414 | 88 (85, 90) | .. | .. | .. | .. | .. | .. | .. | .. | .. | .. | .. | .. | |
| | | | | | BNT162b2+AZD1222 | 1(>21) | Alpha (B.1.1.7) | 2226/53696 | 7313/103684 | 49 (46, 52) | .. | .. | .. | .. | .. | .. | .. | .. | .. | .. | .. | .. | |
| | | | | | | | Delta (B.1.617.2) | 1493/52963 | 4043/100414 | 31 (25, 36) | .. | .. | .. | .. | .. | .. | .. | .. | .. | .. | .. | .. | |
| | | | | | | 2(≥14) | Alpha (B.1.1.7) | 143/24136 | 7313/103684 | 88 (85, 90) | .. | .. | .. | .. | .. | .. | .. | .. | .. | .. | .. | .. | |
| | | | | | | | Delta (B.1.617.2) | 340/24333 | 4043/100414 | 80 (77, 82) | .. | .. | .. | .. | .. | .. | .. | .. | .. | .. | .. | .. | |
