# Supplementary material for: Effectiveness and safety of SARS-CoV-2 vaccine in real-world studies: a systematic review and meta-analysis
Source: Infect Dis Poverty. 2021 Nov 14;10:132. doi: 10.1186/s40249-021-00915-3 (PMC8590867; doi:10.1186/s40249-021-00915-3)
Supplement: Supplementary file 2 — Additional file 2: Table S2. Characteristic of studies included for vaccine safety. [file 40249_2021_915_MOESM2_ESM.pdf]

Table S2. Characteristic of studies included for vaccine safety

First author	Published time	Study design	Study location	Study population	Vaccine type	No. people vaccinated	No. people with adverse reactions	No. people with severe adverse reactions	No. people with vaccine-related death	risk of bias
China CDC	2021.5	cross-sectional	China	general population	Any	265000000	31434	5544	..	moderate
David K. Shay	2021.5	cross-sectional	USA	general population	Ad26.COV2.S	7980000	13725	343	88	moderate
Laith J. Abu-Raddad	2021.6	cross-sectional	Saudi Arabia	general population	BNT162b2	130	52	..	..	moderate
					AZD1222	385	255	..	..	
S Andrzejczak-Grządko	2021.7	cross-sectional	Poland	general population	BNT162b2	196	184	..	..	moderate
						177	175	..	..	
					AZD1222	509	491	..	..	
Aurelie Baldolli	2021.7	cross-sectional	France	general population	BNT162b2	1987	1136	183	..	moderate
				patients with a medical history of COVID-19 disease	BNT162b2	61	45	9	..	
Somy Cherian	2021.6	cross-sectional	India	patients with rheumatic diseases	Any	724	436	..	..	moderate
Patrice Chevallier	2021.6	cohort study	France	allogeneic hematopoietic stem-cells recipients	BNT162b2	94	45	..	..	moderate
Caoilfhionn M Connolly	2021.3	cohort study	USA	patients with rheumatic diseases	Any	325	..	1	..	moderate
Victoria Furer	2021.6	cohort study	Israel	adult patients with autoimmune inflammatory rheumatic diseases	BNT162b2	670	..	2	2	moderate
Julianne Gee	2021.2	cross-sectional			Any	13794904	6994	753	113	moderate
Takanao Hashimoto	2021.6	cross-sectional	Japan	Health Care Workers	BNT162b2	181184	..	37	..	moderate
Gang Lv	2021.5	cross-sectional	USA	general population	Any	6688231	..	55	55	moderate
				long-term care facilities	Any	693246	..	37	37	
Pagotto, V.	2021.6	cross-sectional study	Argentina	healthcare workers	Sputnik V COVID-19 vaccine	683	487	12	..	low
C. Signorelli	2021.6	cross-sectional study	Italy	general population	BNT162b2	37900	142	..	..	moderate

Riad, A.	2021.6	cross-sectional study	Turkey	healthcare workers	CoronaVac	780	487	..	..	low
Quiroga, B.	2021.7	cross-sectional stud	Spanish	nephrologists	BNT162b2	565	422	..	..	low
					mRNA-1273	42	21	..	..	
Vallée, A.	2021.7	cross-sectional study	France	healthcare workers	AZD1222	451	274	..	..	moderate
McMurry, R.	2021.7	cohort study	USA		BNT162b2	51795	..	11	..	low
						39058	..	1	..	
					mRNA-1273	1273	..	2	..	
						11851	..	1	..	
Wang, J.	2021.4	cohort study	China	patients with non-alcoholic fatty liver disease	Beijing Institute of Biological Products Co., Ltd.	381	112	..	..	moderate
Monin, L.	2021.5	cohort study	London	patients with cancer	BNT162b2	140	64	..	..	low
						31	9	..	..	
Ram, R.	2021.7	cohort study	Israeli	patients who underwent either allogeneic HCT or CD19-based chimeric antigen receptor T cell (CART) therapy	BNT162b2	80	11	..	..	moderate
						76	18	..	..	
Zhang, M. X.	2021.5	Cross-Sectional Study	China	healthcare workers	CoronaVac	1526	238	16	..	moderate
						1397	204	..	..	
Rosman, Y.	2021.7	Cross-Sectional Study	USA	patients with mast cell disorders	BNT162b2	26	2	..	..	moderate
Riad, A.	2021.6	Cross-Sectional Study	Germany and Czech Republic	Healthcare Workers	AZD1222	92	87	..	..	moderate
Revon-Riviere, G.	2021.7	cross-sectional study	La Timone, AP-HM	adolescents and young adults with cancer	BNT162b2	13	8	..	..	low
						11	7	..	..	
Peled, Y.	2021.5	cohort study		heart transplant recipients	BNT162b2	77	46	..	..	low
						77	40	..	..	
